# Supplementary material for: Enhanced jet stream waviness induced by suppressed tropical Pacific convection during boreal summer
Source: Nat Commun. 2022 Mar 11;13:1288. doi: 10.1038/s41467-022-28911-7 (PMC8917179; doi:10.1038/s41467-022-28911-7)
Supplement: Supplementary file 1 — Supplementary Information [file 41467_2022_28911_MOESM1_ESM.pdf]

Supplementary material for

Enhanced jet stream waviness induced by suppressed tropical Pacific convection during boreal summer

Xiaoting Sun[1, 2, 3, 4], Qinghua Ding[3,*], Shih-Yu Simon Wang[5], Dániel Topál[3,6,7], Qingquan Li[1, 4], Christopher Castro[8], Haiyan Teng[9], Rui Luo[10], Yihui Ding[4]

[1]Key Laboratory of Meteorological Disaster, Ministry of Education/Joint International Research Laboratory of Climate and Environment Change/Collaborative Innovation Center on Forecast and Evaluation of Meteorological Disasters, Nanjing University of Information Science and Technology, Nanjing 210044, China

[2] Chinese Academy of Meteorological Sciences, Beijing 100081, China

[3]Department of Geography and Earth Research Institute, University of California, Santa Barbara, USA

[4]Laboratory for Climate Studies, National Climate Center, China Meteorological Administration, Beijing, China

[5]Department of Plants, Soils, and Climate, Utah State University, Logan, UT, USA

[6]Institute for Geological and Geochemical Research, Research Centre for Astronomy and Earth Sciences, Eötvös Loránd Research Network, Budapest, Hungary

[7]ELTE Eötvös Loránd University, Doctoral School of Environmental Sciences, Budapest, Hungary

[8]Department of Hydrology Atmospheric Sciences, The University of Arizona, Tucson, Arizona, USA

[9]Pacific Northwest National Laboratory, Richland, WA, USA

[10]Pilot National Laboratory for Marine Science and Technology (Qingdao), Qingdao 266237, China

**Corresponding author address:* Dr. Qinghua Ding, Department of Geography, and Earth Research Institute, University of California, Santa Barbara, Santa Barbara, California, USA. Email: Qinghua@ucsb.edu

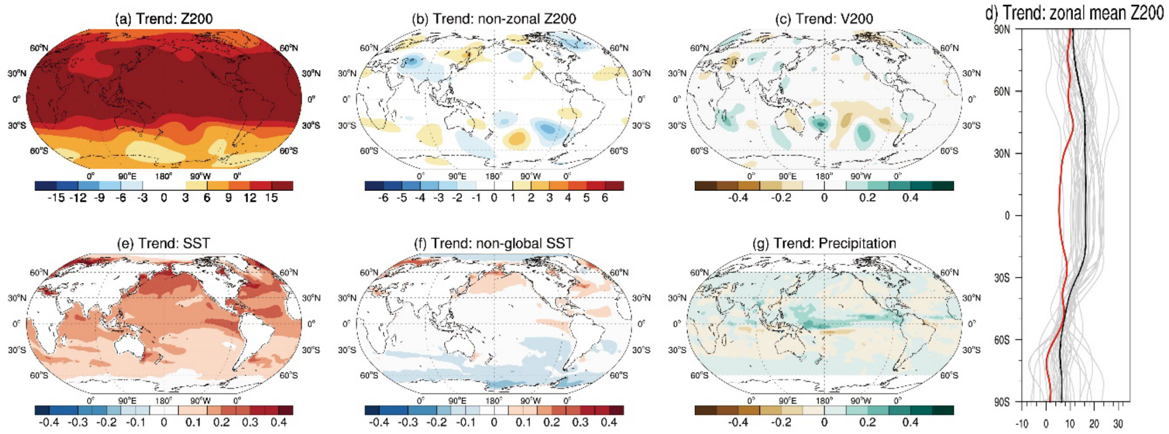

Supplementary Fig. 1: The circulation and SST trends over the past 40 years derived from CMIP5 historical+RCP8.5 experiments

Linear trends (from 1979 to 2018) of JJA (a) Z200 (unit: m/decade) (b) non-zonal Z200 (unit: m/decade) (c) V200 (unit: $m \cdot s^{-1}$/decade) (d) the zonal mean component of Z200 (unit: m/decade) (e) sea surface temperature (SST; unit: K/decade) (f) non-global SST (unit: K/decade) (g) precipitation (unit: mm/day/decade) derived from the ensemble means of CMIP5 historical+RCP8.5 experiments. The grey, black and red lines in (d) represent each of 30 models in CMIP5, the mean of the 30 models and ERA5 reanalysis, respectively.

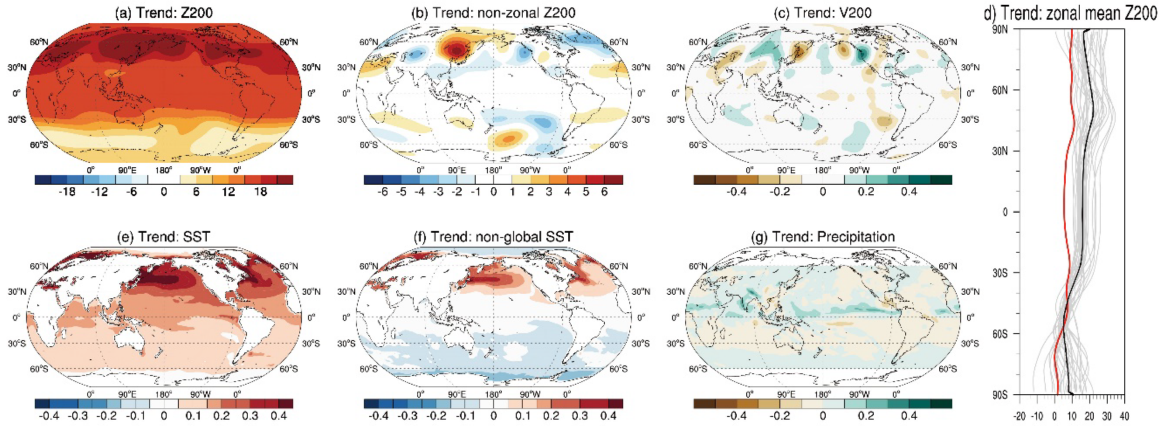

Supplementary Fig. 2: The circulation and SST trends over the past 36 years from 35 CMIP6 climate models

linear trends (from 1979 to 2014) of JJA (a) Z200 (unit: m/decade) (b) non-zonal Z200 (unit: m/decade) (c) V200 (unit: $m\cdot s^{-1}$/decade) (d) the zonal mean component of Z200 (unit: m/decade) (e) sea surface temperature (SST; unit: K/decade) (f) non-global SST (unit: K/decade) (g) precipitation (unit: mm/day/decade) derived from the ensemble means of 35 CMIP6 climate models. The grey, black and red lines in (d) represent each of 35 models in CMIP6, the mean of the 35 models and ERA5 reanalysis, respectively.

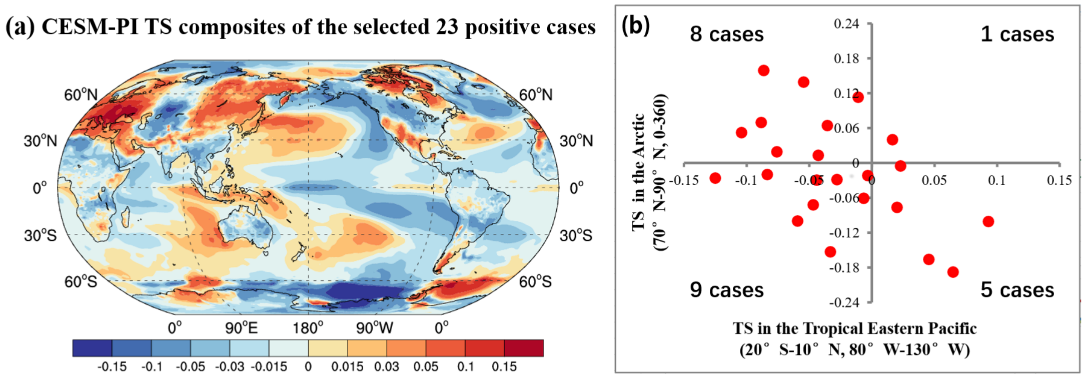

Supplementary Fig. 3: the distribution of the 23 positive events in a scatter plot using the Pan-Arctic average surface temperature (TS) and tropical Eastern Pacific (TEP) average TS as the two coordinates

(a) The composite of surface temperature trends (TS, unit: K/decade) derived from those cases when the pseudo-ensemble (each consecutive 40-yr period) of the CESM-PI run shows the highest spatial correlation (20°N-60°N) with the observed non-zonal Z200 trend (defined as 'the 23 positive cases'). (b) The scatter plot between domain-averaged TS in the tropical Eastern Pacific (20°S-10°N, 80°W-130°W) and domain-averaged TS in the Arctic region (70°N-90°N, 0°-360°) for the selected 23 positive cases.

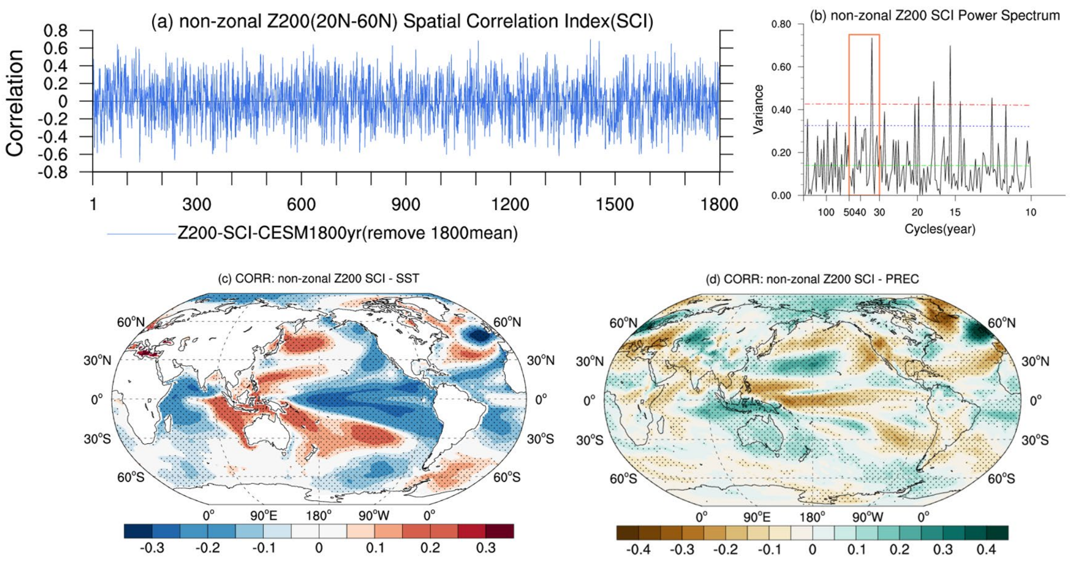

Supplementary Fig. 4: The Z200 Spatial Correlation Index and its correlations with SST and precipitation

(a) Spatial correlations between JJA non-zonal Z200 trend (from 1979-2018) in ERA5 (in Figure 1b) and JJA non-zonal Z200 derived from CESM-LE PI simulation in 1800 years within the mid-high latitude (20°N-60°N), which is defined as Z200 Spatial Correlation Index (SCI). (b) Fourier power spectrum of the non-zonal Z200 SCI (with the cycles longer than 10 years) in (a). The red, blue and green lines in (b) represent 95%, 90% "red noise" confidence and the "red noise" curve. The orange box highlights spectral peaks between 30~50yr. Correlations (CORR) between (c) global SST and (d) precipitation with the Z200 SCI in 1800 years of CESM-LE PI simulations. The dots in (c)-(d) represent correlations coefficients above the 95% confidence level.

The CGT representing an internal circulation mode in the extratropics

If the recent extratropical circulation trend partly results from a low frequency variability, then we should expect to observe a similar mode over a longer period beyond the past 40 years. To place recent years’ atmospheric variability into the context of long-term circulation variability, we perform EOF analysis (Methods) on JJA V200 in the NH (20°N-90°N) in three reanalyses to explore the long-term behaviour of midlatitude circulation over the past 40, 110 and 180 years respectively, considering that the variability of meridional winds around these latitudes can well reflect the waviness of midlatitude zonal flow.

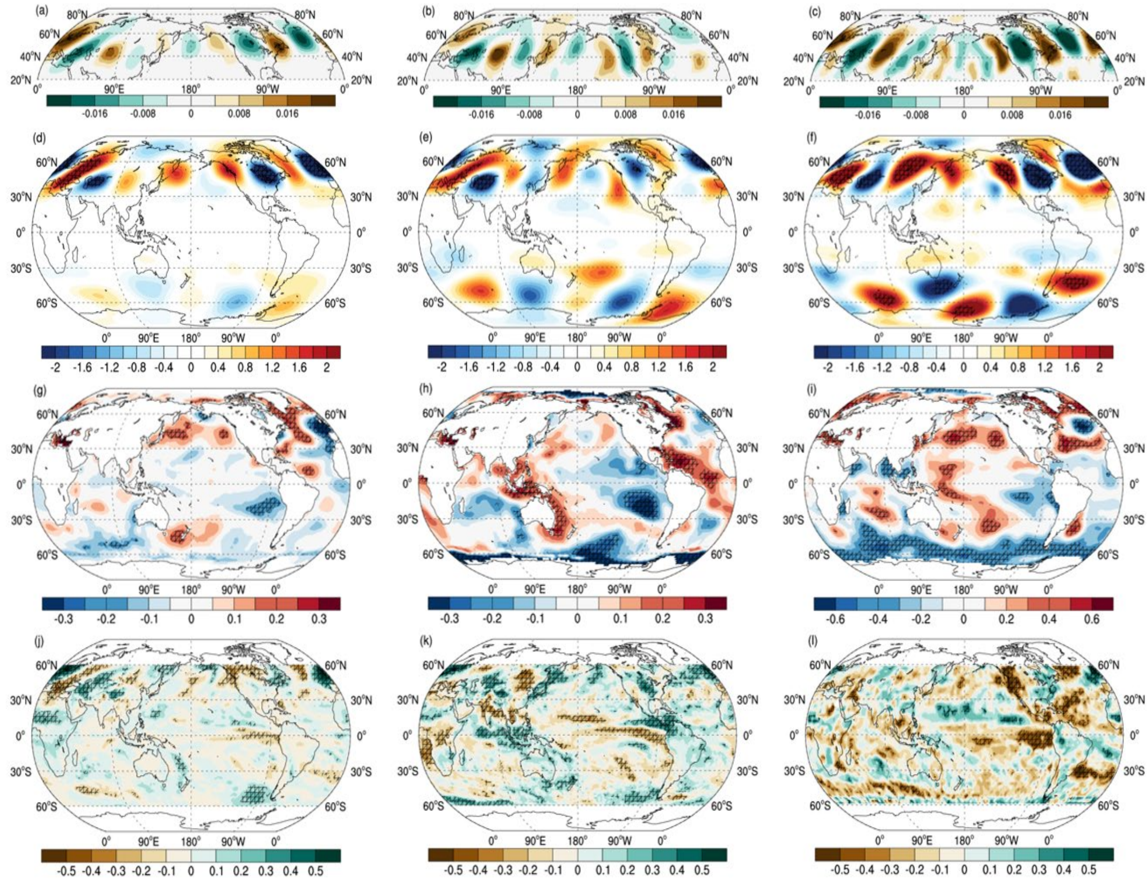

Supplementary Fig. 5: The leading midlatitude circulation variability derived from NOAA-20CR, ERA-20C and ERA5

(a)-(c) The EOF2 mode for JJA V200 in the NH (20°N-90°N), (d)-(f) Regression coefficients between the V200 PC2 and non-zonal Z200, (g)-(i) Correlation coefficients between the V200 PC2 and non-global SST and (j)-(l) Correlation coefficients between the V200 PC2 and precipitation in boreal summer. The cross-hatched areas indicate correlation coefficients above the 95% significance level. The figures on the left, middle and right column are results from NOAA-20CR ((a), (d) and (j): 1836-2015; (g): 1854-2015), ERA-20C ((b), (e), (h) and (k): 1900-2010) and ERA5 ((c), (f), (i) and (l): 1979-2018) reanalysis datasets, respectively.

The first two EOF modes of JJA V200 from all reanalyses display a zonally propagating wave train over longer periods with the second mode showing a striking resemblance to the recent 40 years’ V200 trend (Supplementary Figure 5a-c vs. Figure 1d). The first mode illustrates a different type of wave train propagating along the polar jet at higher latitudes (Figure not shown), that is not the focus of the current study. EOF2 of V200 explains 9.29% 8.19% and 10.13% of the total variance of meridional winds in NOAA-20CR, ERA-20C and ERA5, respectively, and in most regions the maximum V200 amplitude is located within the

jet core with the wave train being almost circumglobal. The regression coefficients between PC2 of V200 and the non-zonal Z200 in the century-long reanalyses exhibit strong similarity to the observed non-zonal Z200 trend over the past 40 years (Figure 1b), altogether showing a well-defined CGT pattern (Supplementary Figure 5d-f). PC2 from the three datasets are highly correlated with each other over their overlapping periods (ERA5 and NOAA-20CR: r = 0.8; ERA5 and ERA-20C: r = 0.47; NOAA-20CR and ERA-20C: r = 0.59) and all exhibit a pronounced low frequency oscillation with a significant positive trend (p<0.01) in the past four decades (Supplementary Fig 6a). This latter is indicative of a gradual enhancement of a CGT like wave train over the recent 40 years. A Fourier power spectrum analysis (Methods) of these PC2 reveals that, the CGT contains a quite broad frequency variability with its interdecadal component characterized by 30-50 years oscillation, in agreement with that found in CESM-LE PI simulation (Supplementary Fig 4b&6b).

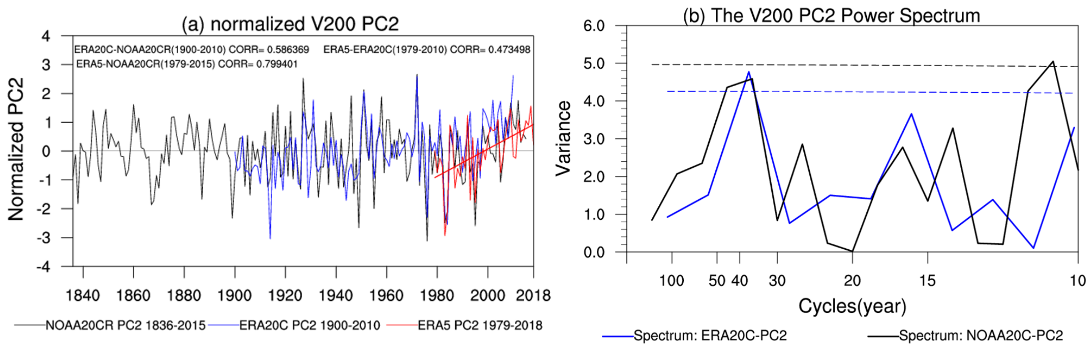

Supplementary Fig. 6: normalized PC2 for JJA V200 and corresponding power spectrum derived from ERA-20C, NOAA-20CR and ERA5 in NH

(a) black, blue and red lines in (a) represent the normalized PC2 for JJA V200 derived from NOAA-20CR, ERA-20C and ERA5 in NH (20°N-90°N), respectively. The thick red line represents a linear trend fit on the ERA5 PC2 time series. The numbers indicate correlation coefficients between any two PCs derived from the reanalysis datasets; (b) black and blue solid lines are fourier power spectrum of normalized V200 PC2 derived from NOAA-20CR and ERA-20C (with the cycles longer than 10 years), respectively. The black and blue dotted lines are 90% and "red noise" confidences derived from NOAA-20CR and ERA-20C, respectively.

The correlation of V200 PC2 with non-global SST (Methods) and precipitation (Supplementary Figure 5g-i) in observations yield significant negative SST cooling and drying along the ITCZ albeit with some structural differences among the three datasets, which is possibly due to the different length of the calculation or large uncertainty of precipitation and SST in the century-long datasets. Nonetheless, the common feature of all tropical SST and precipitation patterns is a suppressed convection surrounding the ITCZ over the TWCP and cooling SST over the TEP. Our observational based results suggest that low frequency variability of tropical SST and precipitation anomalies is closely associated with the midlatitude atmospheric wave pattern captured by the EOF2 of V200 and partially responsible for the recent enhancement in waviness of the midlatitude zonal flow in summer.

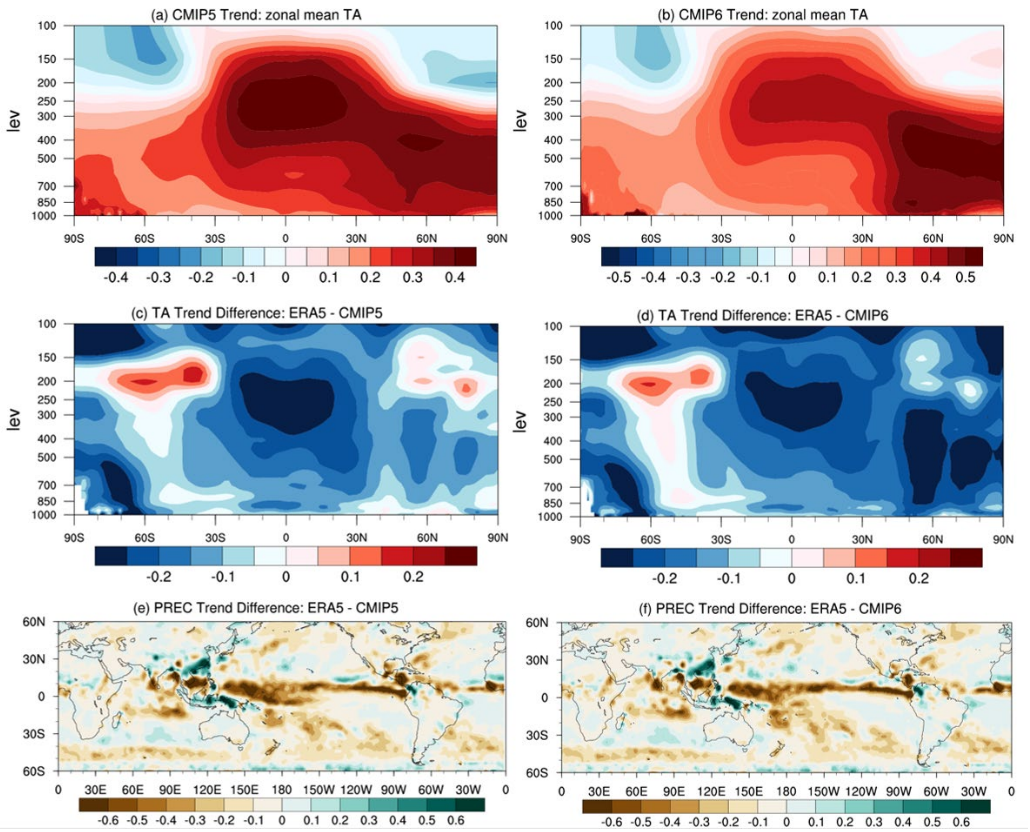

Supplementary Fig. 7: JJA zonal mean component of air temperature (TA) trend profiles
The result is derived from (a) 30 models in CMIP5 historical+RCP8.5 simulations (1979-2018, unit: K/decade) and (b) 35 models in CMIP6 historical forcing (1979-2014, unit: K/decade), respectively. Difference of zonal mean component of TA trends between ERA5 and (c) CMIP5, and between ERA5 and (d) CMIP6. Differences of precipitation (PREC) trends between ERA5 and (e) CMIP5, and between ERA5 and (f) CMIP6. The shaded colors in (e) and (f) indicate differences of precipitation trends between ERA5 and CMIP5/6 (60°S-60°N; unit: mm/day/decade).

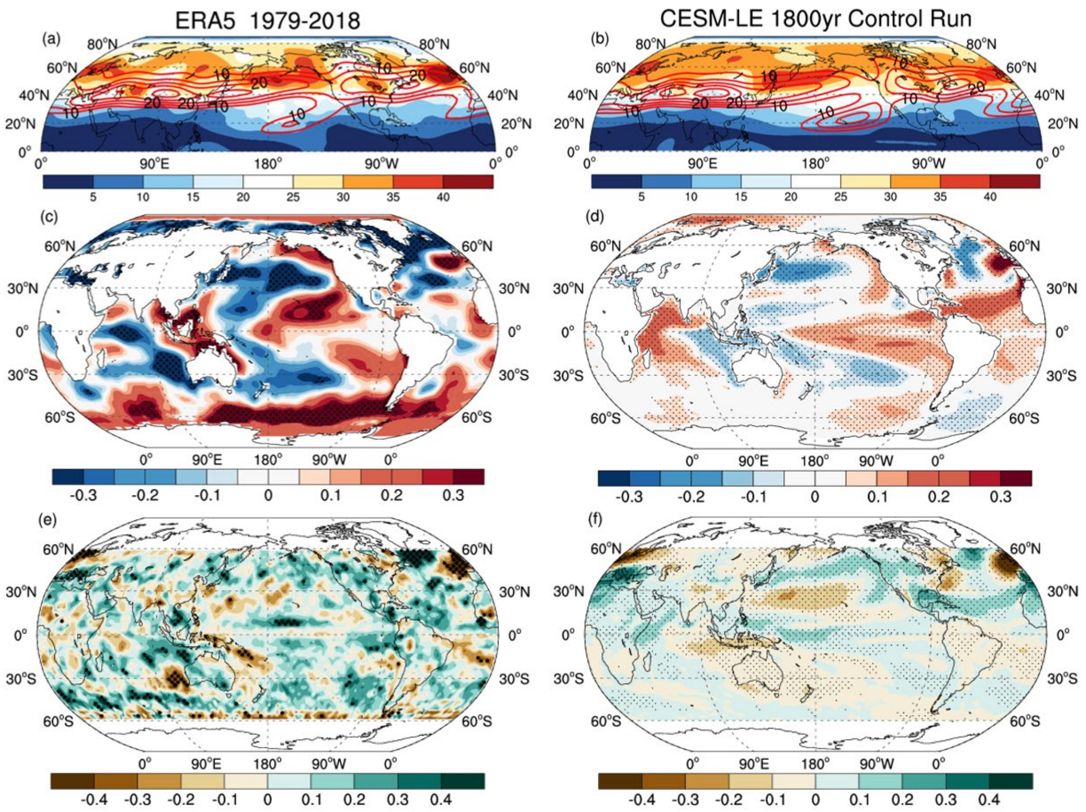

Supplementary Fig. 8: One-point correlations derived from ERA5 and CESM-LE control run

(a, b) Standard deviation of JJA non-zonal Z200 (unit: m) derived from ERA5 and CESM-LE control run, respectively. (c, d) One-point correlation maps between JJA non-zonal Z200 within the jet exit region (JER: 45°N-55°N, 0°-20°W) and global JJA SST. (e, f) One-point correlation maps between JJA non-zonal Z200 over the jet exit region and global precipitation. The cross-hatched areas in (c-f) represent correlation coefficients above the 95% significance level. The red contours in (a) and (b) indicate the climatological 200 hPa zonal wind (unit: m/s), calculated from ERA5 (1979-2018) and CESM-LE PI run respectively.

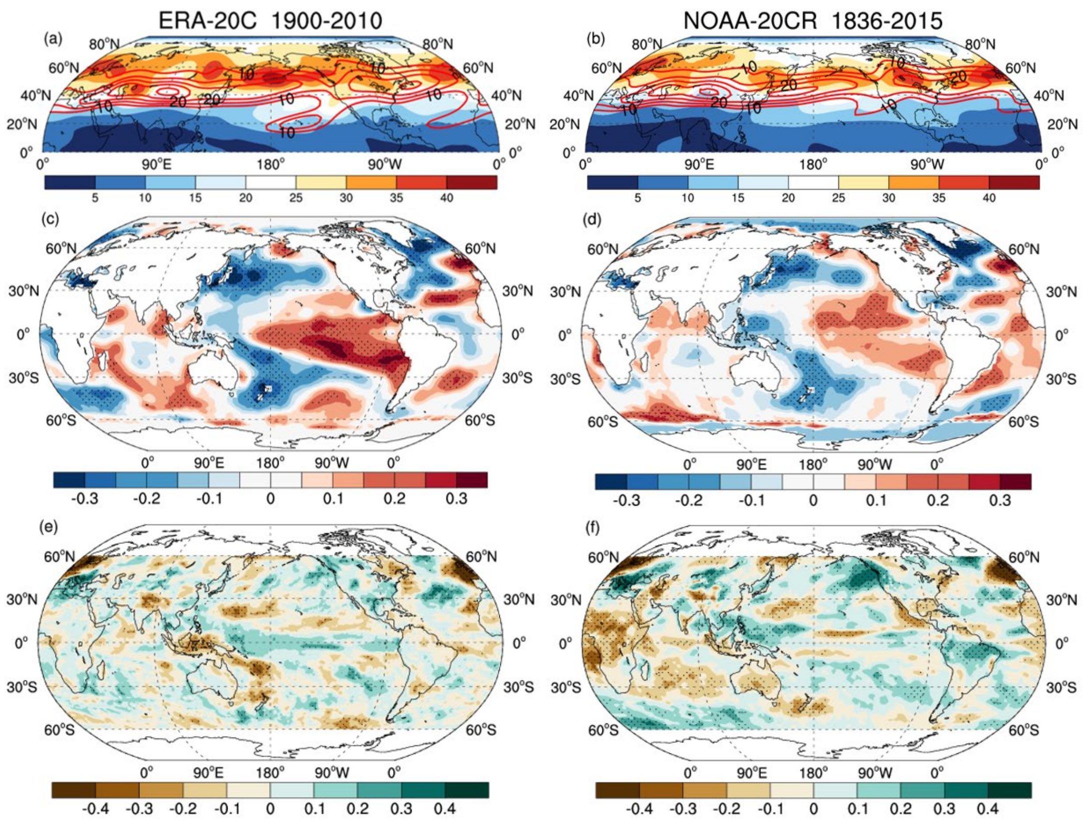

Supplementary Fig. 9: One-point correlations derived from ERA-20C and NOAA-20CR (a, b) Standard deviation of JJA non-zonal Z200 (unit: m) derived from ERA-20C and NOAA-20CR, respectively. (c, d) One-point correlation maps between JJA non-zonal Z200 over the jet exit region (JER: 45°N-55°N, 0°-20°W) and non-global JJA SST. (e, f) One-point correlation maps between JJA non-zonal Z200 over the jet exit region and global JJA precipitation. The cross-hatched areas in (c)-(f) represent correlation coefficients above the 95% significance level.

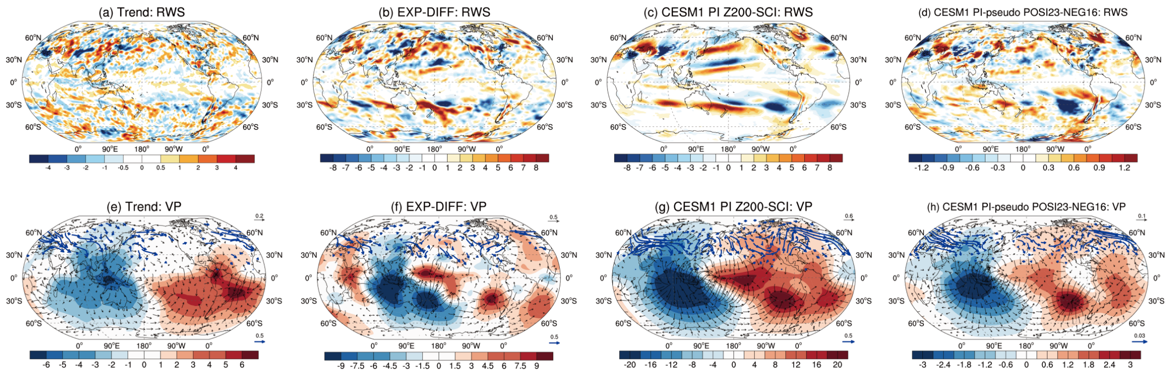

Supplementary Fig. 10: The Rossby wave source, velocity potential, divergent wind component and wave activity fluxes at 200hPa from ERA5, the heating-imposed simulation and pseudo-ensemble of CESM-LE PI runs.
Linear trends of JJA (a) Rossby wave source (RWS; shading; unit: $10^{-11}s^{-2}$/decade) (e) velocity potential (VP; shading; unit: 10^{5} m^{2}/s/decade), divergent wind component (black vectors; unit: $m \cdot s^{-1}$/decade) and wave activity fluxes (WAF; in the Northern Hemisphere; blue vectors; unit: m^{2}/s^{2}/decade) over the past 40 years from ERA5. (b, f) are results from simulated response of JJA RWS (unit: $10^{-11}s^{-2}$), VP (unit: 10^{5} m^{2}/s), divergent wind component (unit: $m \cdot s^{-1}$) and WAF (unit: m^{2}/s^{2}) to anomalous tropical heating sources added in the CESM derived from Fig. 6 (the difference of SEN and CTL). (c, g) are from regression between the Z200 SCI and RWS/VP in 1800 years of CESM-LE PI simulations. (d, h) are from differences of RWS/VP between the selected 23 positive and 16 negative cases in the pseudo-ensemble of CESM-LE PI runs, respectively.

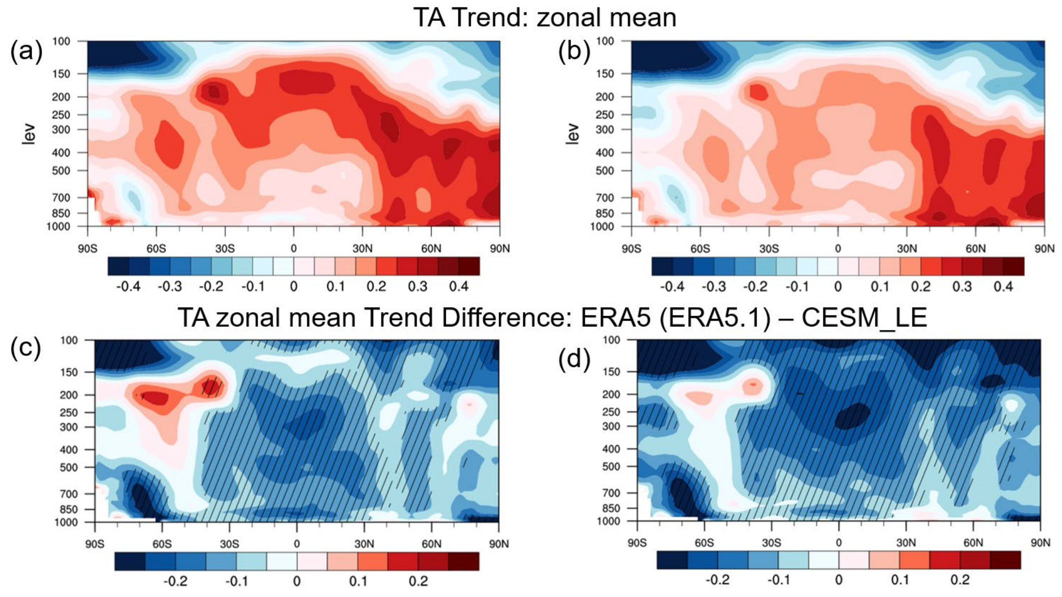

Supplementary Fig. 11: Air temperature (TA) trends from ERA5+ERA5.1 and ERA5-only. Linear trends of JJA zonal mean TA derived from (a) ERA5 and ERA5.1 (ERA5 over 2000-2006 is replaced by ERA5.1, unit: K/decade) and (b) ERA5-only, respectively. Differences of zonal mean TA trends between (c) ERA5 and ERA5.1, (d) ERA5-only and the ensemble mean of CESM-LE 40-member historical runs. The cross-hatched areas indicate the ERA5 and ERA5.1 (ERA5-only) trends lie outside the 2 standard deviations from the ensemble mean of CESM-LE 40-member historical runs. The (b) and (d) are also shown as Fig. 5 (a) and (c).

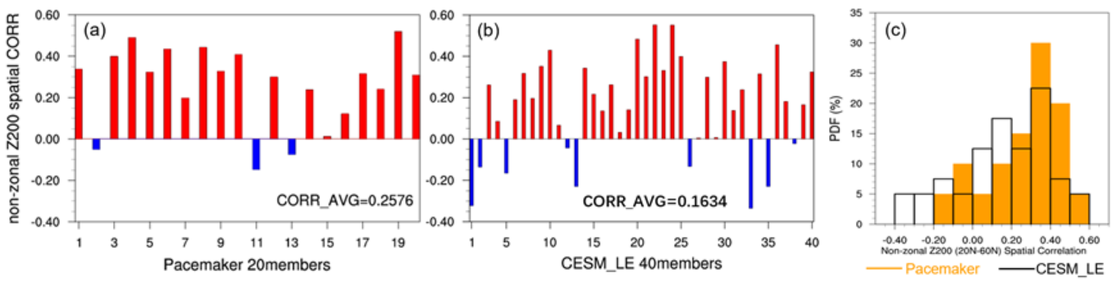

Supplementary Fig. 12: The spatial correlations between non-zonal JJA Z200 trends derived from ERA5 and CESM1 Pacemaker/CESM-LE simulations.
The spatial correlations between non-zonal JJA Z200 trends derived from ERA5 and (a) CESM1 Pacific Pacemaker 20-member simulations (1979-2013), and (b) CESM-LE 40-member historical simulations within the mid-high latitudes (20°N-60°N), respectively. (c) The probability density functions (PDF) of spatial correlations from (a) and (b) are calculated to indicate an improvement of the Pacemaker runs (yellow bars) in simulating the non-zonal JJA Z200 trend pattern in ERA5 over that of CESM-LE (black bars).

Supplementary Table 1: 30 climate models in CMIP5 historical+RCP8.5 simulations and 35 models in CMIP6 historical simulations that are used in the study

CMIP5 model name	*CMIP6 model name*
1. ACCESS1-0	*1. ACCESS-CM2*
2. ACCESS1-3	*2. ACCESS-ESM1-5*
3. CanESM2	*3. AWI-CM-1-1-MR*
4. CMCC-CESM	*4. AWI-ESM-1-1-LR*
5. CMCC-CM	*5. BCC-CSM2-MR*
6. CMCC-CMS	*6. BCC-ESM1*
7. CNRM-CM5	*7. CanESM5*
8. CSIRO-Mk3.6.0	*8. CESM2-FV2*
9. FGOALS-s2	*9. CESM2-WACCM-FV2*
10. GFDL-CM3	*10. CESM2-WACCM*
11. GFDL-ESM2G	*11. CESM2*
12. GFDL-ESM2M	*12. CIESM*
13. GISS-E2-H	*13. E3SM-1-0*
14. GISS-E2-H-CC	*14. E3SM-1-1-ECA*
15. GISS-E2-R	*15. E3SM-1-1*
16. GISS-E2-R-CC	*16. EC-Earth3-Veg*
17. HadGEM2-AO	*17. EC-Earth3*
18. HadGEM2-CC	*18. FGOALS-f3-L*
19. HadGEM2-ES	*19. FGOALS-g3*
20. INM-CM4	*20. FIO-ESM-2-0*
21. IPSL-CM5B-LR	*21. GFDL-ESM4*
22. MIROC-ESM	*22. INM-CM4-8*
23. MIROC-ESM-CHEM	*23. INM-CM5-0*
24. MIROC5	*24. IPSL-CM6A-LR*
25. MPI-ESM-LR	*25. MCM-UA-1-0*
26. MPI-ESM-MR	*26. MIROC6*
27. MRI-CGCM3	*27. MPI-ESM-1-2-HAM*
28. MRI-ESM1	*28. MPI-ESM1-2-HR*
29. NorESM1-M	*29. MPI-ESM1-2-LR*
30. NorESM1-ME	*30. MRI-ESM2-0*
	31. NESM3
	32. NorCPM1
	33. NorESM2-LM
	34. NorESM2-MM
	35. SAM0-UNICON
